# Membrane-initiated estrogen receptor-α signaling in osteoblasts is crucial for normal regulation of the cortical bone in female mice

**DOI:** 10.1038/s41413-025-00439-8

**Published:** 2025-06-17

**Authors:** Yiwen Jiang, Karin Horkeby, Petra Henning, Jianyao Wu, Karin H. Nilsson, Lina Lawenius, Sofia Movérare-Skrtic, Priti Gupta, Cecilia Engdahl, Antti Koskela, Juha Tuukkanen, Lei Li, Claes Ohlsson, Marie K. Lagerquist

**Affiliations:** 1 Sahlgrenska Osteoporosis Centre, Centre for Bone and Arthritis Research, Institute of Medicine, Sahlgrenska Academy at University of Gothenburg, S-41346 Gothenburg, Sweden; 2 Department of Rheumatology and Inflammation Research, Institute of Medicine, Sahlgrenska Academy at University of Gothenburg, S-41346 Gothenburg, Sweden; 3 Department of Anatomy and Cell Biology, Faculty of Medicine, Translational Medicine Research Unit, University of Oulu, FI-90014 Oulu, Finland; 4 Department of Drug Treatment, Sahlgrenska University Hospital, Region Västra Götaland, S-41346 Gothenburg, Sweden

**Keywords:** Bone, Endocrine system and metabolic diseases

## Abstract

Membrane-initiated estrogen receptor α (mERα) signaling has been shown to affect bone mass in murine models. However, it remains unknown which cell types mediate the mERα-dependent effects on bone. In this study, we generated a novel mouse model with a conditional C451A mutation in *Esr1*, which enables selective knockout of the palmitoylation site essential for the membrane localization of ERα (C451A^f/f^). First, we used *Runx2*-Cre mice to generate *Runx2*-C451A^f/f^ mice with conditional inactivation of mERα signaling in *Runx2*-expressing osteoblast lineage cells. No significant changes were observed in body weight, weights of estrogen-responsive organs, or serum concentrations of estradiol between female *Runx2*-C451A^f/f^ and homozygous C451A^f/f^ littermate controls. High-resolution microcomputed tomography analysis showed a consistent decrease in cortical bone mass in the tibia, femur, and vertebra L5 of *Runx2*-C451A^f/f^ mice and three-point bending analysis of humerus revealed an impaired mechanical bone strength in *Runx2*-C451A^f/f^ female mice compared to controls. Additionally, primary osteoblast cultures from mice lacking mERα signaling showed impaired differentiation compared to controls. In contrast, conditional inactivation of mERα signaling in hematopoietic cells, by transplantation of bone marrow from mice lacking mERα signaling in all cells to adult wildtype female mice, did not result in any skeletal alterations. In conclusion, this study demonstrates that mERα signaling in osteoblast lineage cells plays a crucial role in the regulation of cortical bone in female mice and shows that mERα inactivation in hematopoietic cells of adult female mice is dispensable for bone regulation.

## Introduction

Estrogen plays an important role in maintaining healthy bone metabolism. The association between estrogen deficiency and osteoporosis is well-established in both humans^1–4^ and animal models.^5–7^ However, the pleiotropic effects of estrogen pose challenges in the prevention and treatment of osteoporosis by hormone replacement therapy, as it may result in side effects in other tissues, such as cancer in reproductive organs and thromboembolism.^8–10^ To be able to preserve the protective effects of estrogen against bone loss with minimal effects on other tissues, we need to better understand the regulation of estrogen effects in various tissues and cell types.

Estrogen effects are mainly mediated by estrogen receptor alpha and beta (ERα, β), which are expressed in many cell types throughout the body.^11^ The estrogen protection against bone loss is primarily mediated via ERα signaling.^12–14^ ERα is expressed in different cell types within the bone tissue,^15^ eliciting various functions upon activation to regulate bone remodeling. Previous animal studies have shown that deletion of ERα signaling in osteoblast progenitor cells results in reduced cortical bone mass, while loss of ERα signaling in osteoclasts causes trabecular bone loss in female mice.^16–18^ The role of ERα signaling in mature osteoblasts and osteocytes remains uncertain based on conflicting results from different studies.^18–21^

Previous work by us and others has demonstrated that, in addition to the role of ERα as a transcription factor, rapid membrane-initiated ERα (mERα) signaling also contributes significantly to the bone-sparing effect of estrogen.^22–26^ Inactivation of mERα signaling in mice is accomplished by introducing an amino acid shift at site 451 (C451A) in *Esr1*, thereby disrupting the essential palmitoylation of ERα required for membrane localization.^27,28^ The mERα-dependent estrogen effects have been shown to be tissue-specific,^22,25–28^ however, the specific cell types responsible for the mERα-dependent effects of estrogen on bone remain unknown. We have therefore generated a conditional mERα signaling knock-out model with the aim to investigate the role of mERα signaling in specific cell types for the regulation of bone mass.

## Results

### Loss of mERα signaling in *Runx2*-C451A female mice is restricted to bone tissue

**Fig. 1:**
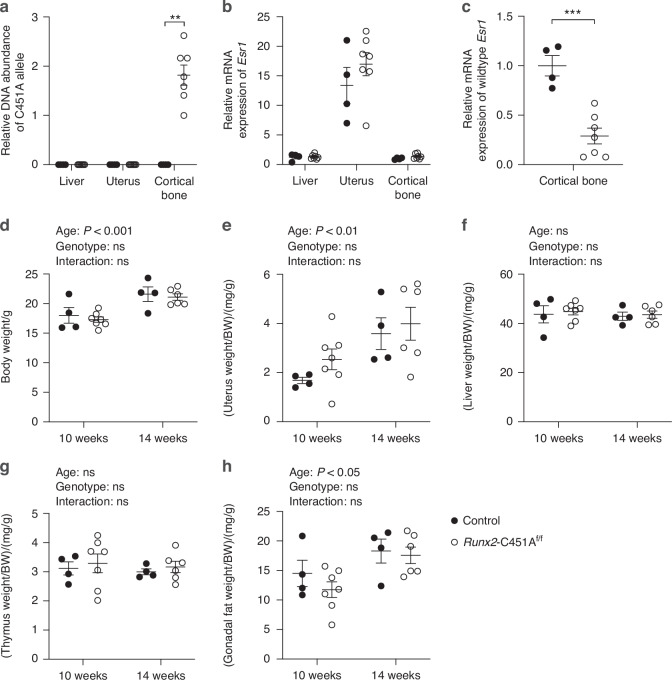
Validation of the *Runx2-*C451A model. Female *Runx2*-C451A^f/f^ and homozygous C451A^f/f^ littermates were terminated at 10 and 14 weeks of age. **a** DNA analysis at 10 weeks of age showing abundance of the mutated allele in liver, uterus, and cortical bone related to the sample with the lowest detectable abundance in the *Runx2*-C451A^f/f^ group in cortical bone. Total mRNA expression of *Esr1* (including both the wildtype and mutated *Esr1*) at 10 weeks of age in liver, uterus, and cortical bone (**b**), and mRNA expression of wildtype *Esr1* in cortical bone at 10 weeks of age (**c**), related to the expression in the control group in cortical bone. Body weight (**d**), and the body weight-related weights of estrogen-responsive organs, including uterus (**e**), liver (**f**), thymus (**g**), and gonadal fat (**h**) at 10 and 14 weeks of age. Mann–Whitney *U* test was applied to analyze the difference of DNA abundance of the C451A allele (**a**), and Student’s *t* test was applied to analyze the relative mRNA expression of *Esr1* (**b**, **c**). **d**–**h** Two-way ANOVA test was applied for the weight analyses. All individual values are presented with mean (horizontal line) and SEM (vertical lines). **P < 0.01, ***P < 0.001, ns not significant, BW body weight

To determine the role of mERα signaling in specific cells, we generated a mouse model with a conditional C451A mutation in *Esr1* (C451A^f/f^ mice). *Runx2*-Cre mice were used to generate *Runx2*-C451A^f/f^ mice with conditional inactivation of mERα signaling in osteoblast lineage cells, and homozygous C451A^f/f^ littermates were used as controls. DNA analysis showed presence of the mutated C451A-*Esr1* allele in bone tissue of *Runx2*-C451A^f/f^ mice, while the mutation was not detected in liver or uterus (Fig. 1a). The total mRNA expression of *Esr1*, from both the wildtype (WT) and the mutated allele, remained similar between *Runx2*-C451A^f/f^ and control mice in liver, uterus, and cortical bone (Fig. 1b), while the mRNA expression of WT *Esr1* decreased by 71% in the cortical bone of *Runx2*-C451A^f/f^ compared to control mice (Fig. 1c). Body weight, along with body weight-related weights of estrogen-responsive organs such as the uterus, liver, thymus, and gonadal fat, was not changed between *Runx2*-C451A^f/f^ and control mice (Fig. 1d–h). No significant alterations were observed between *Runx2*-C451A^f/f^ and controls when measuring the serum concentrations of estradiol (E2) and testosterone (T) (Table 1).

**Table 1:** Circulating levels of estradiol and testosterone

		Control	*Runx2*-C451A^f/f^
10-week-old	Estradiol/(pg/mL)	1.2 ± 0.8	3.3 ± 5.9
	Testosterone/(pg/mL)	21.8 ± 5.2	45.5 ± 27.2
14-week-old	Estradiol/(pg/mL)	3.9 ± 4.7	16.7 ± 11.6
	Testosterone/(pg/mL)	21.0 ± 4.3	29.0 ± 11.0

Serum concentrations of estradiol and testosterone were measured in female mice, by high-sensitivity liquid chromatography-tandem mass spectrometry at the age of 10 weeks (Control, *n* = 4; *Runx2*-C451A^f/f^, *n* = 7) and 14 weeks (Control, *n* = 4; *Runx2*-C451A^f/f^, *n* = 4). Mann–Whitney U test was applied. All values are presented as mean ± SD

### The absence of mERα signaling in *Runx2* osteoblast lineage cells leads to reduced bone mass and mechanical strength

**Fig. 2:**
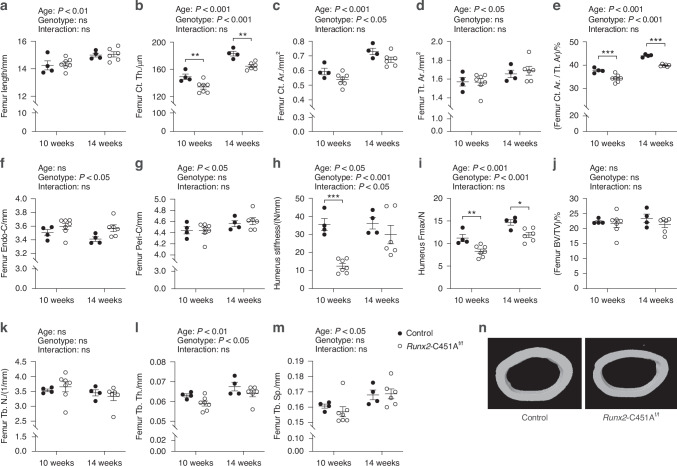
Loss of mERα signaling in osteoblast lineage cells reduces bone mass and mechanical strength. Bone parameters of the femur were measured in female C451A^f/f^ and *Runx2-*C451A^f/f^ mice by high-resolution microcomputed tomography at the age of 10 weeks and 14 weeks, including femur length (**a**), cortical thickness; Ct. Th. (**b**), cortical area; Ct. Ar. (**c**), total cross-sectional area; Tt, Ar. (**d**), cortical fraction area; Ct. Ar./Tt. Ar. (**e**), endosteal circumference; Endo-C (**f**), periosteal circumference; Peri-C (**g**), and trabecular bone volume fraction; BV/TV (**j**), trabecular number; Tb. N. (**k**), trabecular thickness; Tb. Th. (**l**), trabecular separation; Tb. Sp. (**m**), representative pictures of cortical bone (**n**). Bone stiffness (**h**) and maximum force at fracture (**i**) were measured in humerus by three-point bending. Two-way ANOVA test was applied (results presented above each plot) followed by Šidák´s multiple comparisons test to compare difference between genotypes within each time point. A significant difference by Šidák´s multiple comparisons test is indicated by *P < 0.05, **P < 0.01, ***P < 0.001 in the graphs. All individual values are presented with mean (horizontal line) and SEM (vertical lines). ns not significant

There were no differences in the lengths of femur and tibia, or the height of vertebra, between *Runx2*-C451A^f/f^ and control female mice at 10 and 14 weeks of age (Fig. 2a, Table 2). A consistent reduction in cortical thickness and cortical area was demonstrated in the femur, tibia, and L5 of *Runx2*-C451A^f/f^ female mice compared to controls (Fig. 2b, c, n, Table 2), indicating a robust regulation of cortical bone mass via mERα signaling in osteoblast lineage cells. In addition, cortical area fraction was also reduced in femur and tibia (Fig. 2e, Table 2). Notably, a significant increase was observed in the endosteal circumference of both the femur and tibia in *Runx2*-C451A^f/f^ female mice, while the periosteal circumference remained unchanged between *Runx2*-C451A^f/f^ and control mice (Fig. 2f–g, Table 2). Three-point bending analysis of the humerus showed reduced stiffness and maximum force at fracture in *Runx2*-C451A^f/f^ mice compared to control mice, demonstrating impaired mechanical strength (Fig. 2h, i).

**Table 2:** Measurement of bone parameters in the tibia and vertebra L5

		10-week-old		14-week-old		Two-way ANOVA		
		Control	*Runx2-*C451A^f/f^	Control	*Runx2-*C451A^f/f^	Age	Genotype	Interaction
Tibia	Length/mm	16.8 ± 0.3	16.5 ± 0.4	17.7 ± 0.4	17.3 ± 0.2	*P* < 0.001	ns	ns
	(BV/TV)/%	19.3 ± 3.0	14.6 ± 2.8*	22.6 ± 1.5	15.6 ± 1.1***	ns	*P* < 0.001	ns
	Tb. N. /(1/mm)	3.4 ± 0.3	2.8 ± 0.5*	3.3 ± 0.1	2.6 ± 0.2*	ns	*P* < 0.01	ns
	Tb. Th. /mm	0.056 ± 0.005	0.051 ± 0.002	0.068 ± 0.004	0.060 ± 0.004*	*P* < 0.001	*P* < 0.01	ns
	Tb. Sp. /mm	0.162 ± 0.005	0.169 ± 0.012	0.169 ± 0.006	0.181 ± 0.005	*P* < 0.05	*P* < 0.05	ns
	Ct. Th. /μm	185 ± 13	158 ± 11**	211 ± 7	187 ± 5**	*P* < 0.001	*P* < 0.001	ns
	Ct. Ar. /mm^2^	0.62 ± 0.08	0.56 ± 0.05	0.70 ± 0.04	0.64 ± 0.03	*P* < 0.01	*P* < 0.05	ns
	Tt. Ar. /mm^2^	1.24 ± 0.14	1.31 ± 0.08	1.25 ± 0.06	1.28 ± 0.06	ns	ns	ns
	(Ct. Ar./Tt. Ar.)/%	50.2 ± 0.8	43.0 ± 1.7***	55.7 ± 1.2	50.0 ± 0.8***	*P* < 0.001	*P* < 0.001	ns
	Endo-C/mm	2.8 ± 0.1	3.1 ± 0.1	2.6 ± 0.1	2.8 ± 0.1	*P* < 0.001	*P* < 0.001	ns
	Peri-C/mm	4.0 ± 0.2	4.1 ± 0.1	4.0 ± 0.1	4.0 ± 0.1	ns	ns	ns
L5	Height/mm	3.1 ± 0.1	3.2 ± 0.1	3.4 ± 0.2	3.4 ± 0.1	*P* < 0.01	ns	ns
	(BV/TV)/%	17.3 ± 1.7	16.1 ± 2.1	21.5 ± 1.7	18.5 ± 2.0	*P* < 0.01	ns	ns
	Tb. N. /1/mm	3.7 ± 0.3	3.5 ± 0.4	3.8 ± 0.2	3.6 ± 0.4	ns	ns	ns
	Tb. Th. /mm	0.047 ± 0.001	0.045 ± 0.002	0.056 ± 0.002	0.051 ± 0.002**	*P* < 0.001	*P* < 0.01	ns
	Tb. Sp. /mm	0.172 ± 0.005	0.162 ± 0.003	0.171 ± 0.002	0.167 ± 0.008	ns	*P* < 0.05	ns
	Ct. Th. /μm	61.0 ± 4.8	54.1 ± 2.4	73.5 ± 5.7	61.3 ± 4.1	*P* < 0.001	*P* < 0.001	ns
	Ct. Ar. /mm^2^	0.31 ± 0.03	0.26 ± 0.02	0.38 ± 0.04	0.32 ± 0.03*	*P* < 0.001	*P* < 0.01	ns

Bone parameters of the tibia and vertebra L5 from female mice were measured by high-resolution microcomputed tomography at the age of 10 weeks (Control, *n* = 4 for tibia, *n* = 3 for L5; *Runx2*-C451A^f/f^, *n* = 7) and 14 weeks (Control, *n* = 4; *Runx2*-C451A^f/f^, *n* = 6). Two-way ANOVA test was applied followed by Šidák´s multiple comparisons test to compare difference between genotypes within each time point. All values are presented as mean ± SD

*BV/TV* trabecular bone volume fraction, *Tb. N.* trabecular number, *Tb. Th.* trabecular thickness, *Tb. Sp.* trabecular separation, *Ct. Th.* cortical thickness, *Ct. Ar.* cortical area, *Tt, Ar.* total cross-sectional area, *Ct. Ar./Tt. Ar.* cortical fraction area, *Endo-C* endosteal circumference, *Peri-C* periosteal circumference, *ns* not significant

A significant difference by Šidák´s multiple comparisons test is indicated by *P < 0.05, **P < 0.01, ***P < 0.001

Neither trabecular bone volume fraction (BV/TV), reflecting the overall trabecular bone mass, nor trabecular number of femur (Fig. 2j, k) or L5 (Table 2), were significantly affected in *Runx2*-C451A^f/f^ compared to control mice. However, a minor reduction in trabecular thickness was observed in both femur and L5 (Fig. 2l, Table 2). In tibia, trabecular BV/TV, number, and thickness were decreased in the tibia of *Runx2*-C451A^f/f^ mice compared to controls (Table 2).

Dynamic histomorphometry analyses, performed in 10 and 14-week-old *Runx2*-C451A^f/f^ and control mice, did not reveal any effects on bone formation parameters (mineralizing surface/bone surface, mineral apposition rate, bone formation rate/bone surface) at the endosteal or the periosteal surfaces of the mid-diaphysis of femur (Table 3). Serum analyses of bone formation (P1NP) and bone resorption (CTX-I) markers showed no differences between *Runx2*-C451A^f/f^ mice and controls (Table 3).

**Table 3:** Dynamic histomorphometry of the femur and serum markers of bone formation and resorption

		10-week-old		14-week-old	
		Control	*Runx2-*C451A^f/f^	Control	*Runx2-*C451A^f/f^
Endocortical surface	(MS/BS)/%	65.6 ± 3.9	66.4 ± 10.9	79.3 ± 6.0	66.7 ± 11.5
	MAR /(μm/d)	1.07 ± 0.07	1.06 ± 0.25	1.16 ± 0.14	1.13 ± 0.20
	(BFR/BS) (μm^3^/μm^2^/y)	256 ± 19	263 ± 33	338 ± 60	275 ± 63
Periosteal surface	(MS/BS) /%	42.5 ± 7.8	53.9 ± 14.6	48.5 ± 6.5	54.4 ± 9.8
	MAR/ (μm/d)	0.94 ± 0.12	1.05 ± 0.17	1.28 ± 0.24	1.09 ± 0.26
	(BFR/BS)/ (μm^3^/μm^2^/y)	146 ± 35	201 ± 43	232 ± 73	221 ± 84
Serum markers	P1NP/ (ng/mL)	64.8 ± 20.2	71.5 ± 19.2	54.1 ± 22.9	55.7 ± 14.7
	CTX-I/ (ng/mL)	20.2 ± 0.6	34.9 ± 24.1	12.9 ± 3.3	16.7 ± 4.1

Dynamic histomorphometry of the femur and serum markers for bone formation and resorption in female mice at the age of 10 weeks (Control, *n* = 4; *Runx2*-C451A^f/f^, *n* = 7) and 14 weeks (Control, *n* = 4; *Runx2*-C451A^f/f^, *n* = 6). Two-way ANOVA test was applied followed by Šidák´s multiple comparisons test, and no significant differences were detected. All values are presented as mean ± SD

*MS/BS* Mineralizing surface/bone surface, *MAR* Mineral apposition rate, *BFR/BS* Bone formation rate/bone surface, *P1NP* procollagen type I N propeptide, *CTX-I* C-terminal type I collagen fragments

### Loss of mERα signaling in osteoblasts affects transcription of genes related to osteoblast differentiation

**Fig. 3:**
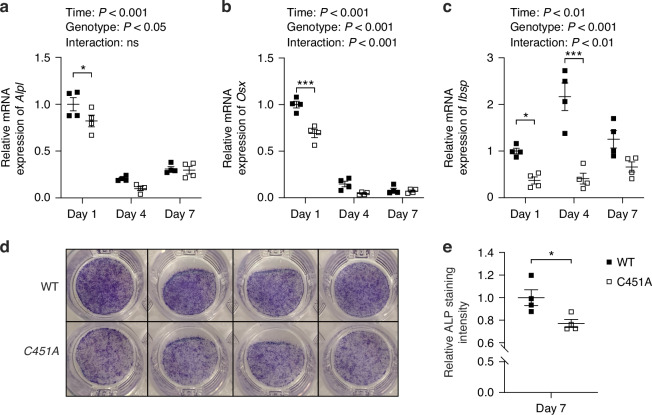
Lack of mERα signaling reduces the expression of genes related to maturation and differentiation in primary osteoblast cultures. The mRNA expression of alkaline phosphatase (*Alpl*, **a**), osterix (*Osx*, **b**), and integrin-binding bone sialoprotein (*Ibsp*, **c**) in primary calvarial osteoblast cultures from 3 to 5 days old WT or global C451A female mice at day 1, 4, and 7, related to the expression in the control group at day 1. ALP-stained primary osteoblasts after 7 days in culture (**d**), and quantification of ALP-staining, related to the control group (**e**). **a**–**c** Two-way ANOVA test was applied (results presented above each plot) followed by Šidák´s multiple comparisons test to compare difference between genotypes within each time point. A significant difference by Šidák´s multiple comparisons test is indicated by *P < 0.05, ***P < 0.001 in the graphs. **e** Student’s *t* test was applied, *P < 0.05, C451A vs. WT calvarial osteoblast cultures. All individual values are presented with mean (horizontal line) and SEM (vertical lines)

Gene transcription analysis of primary osteoblast cultures from global C451A mice, lacking mERα signaling in all cells, and WT mice showed a significant genotype effect with decreased expression of alkaline phosphatase (*Alpl*), osterix (*Osx*), and integrin-binding bone sialoprotein (*Ibsp*) (Fig. 3a–c). ALP staining after 7 days of culturing also showed less ALP-positive osteoblasts in the C451A primary osteoblast cultures compared to the WT controls (Fig. 3d, e).

### Inactivation of mERα signaling in hematopoietic cells does not affect the skeleton

**Fig. 4:**
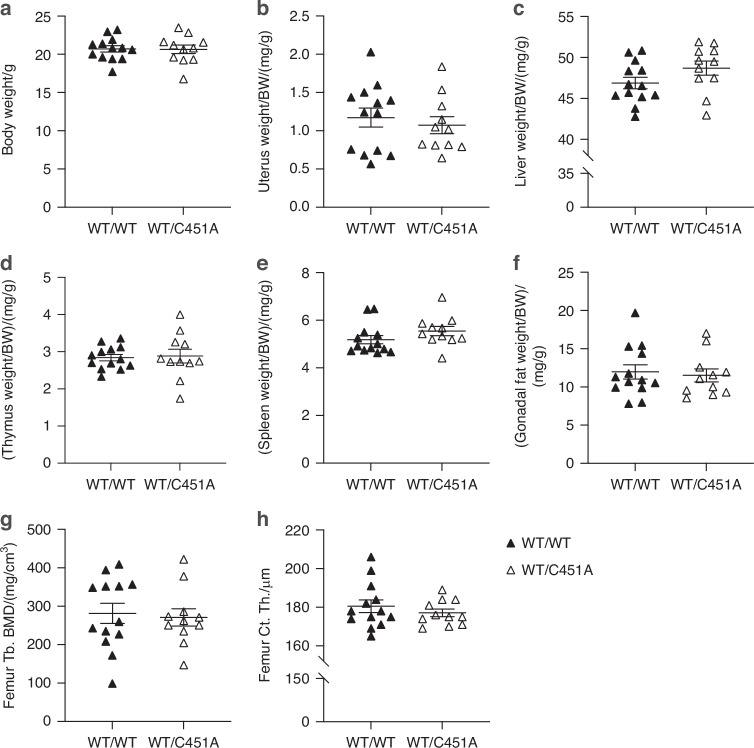
Loss of mERα signaling in hematopoietic cells does not cause significant phenotypic changes in bone or other evaluated organs. Body weights (**a**) and body weight-related organ weights of wildtype (WT) recipient mice receiving WT bone marrow (WT/WT) and WT mice receiving bone marrow from global C451A female mice, lacking mERα signaling in all cells (WT/C451A), including uterus (**b**), liver (**c**), thymus (**d**), spleen (**e**), and gonadal fat (**f**). Femur trabecular bone mineral density; Tb. BMD (**g**) and cortical thickness; Ct. Th. (**h**) were measured by peripheral quantitative computed tomography. Student’s *t* test was applied. All individual values are presented with mean (horizontal line) and SEM (vertical lines). BW body weight

Bone marrow transplantation (BMT) was used to inactivate mERα signaling in hematopoietic cells in 11-week-old female mice. DNA analysis revealed detection of the mutated C451A-*Esr1* allele specifically in the bone marrow of WT mice receiving bone marrow from global C451A mice (WT/C451A), while the mutation was not detected in WT mice receiving bone marrow from WT mice (WT/WT) (Fig. S1). Analyses of WT/WT and WT/C451A mice five weeks after BMT showed no differences in body weight or body weight-related weights of organs including uterus, liver, thymus, spleen, and gonadal fat (Fig. 4a–f). Skeletal analyses revealed that both trabecular and cortical bone in femur, tibia and vertebra were similar between WT/WT and WT/C451A mice (Fig. 4g, h, Table S1), demonstrating that inactivation of mERα signaling in hematopoietic cells of adult female mice does not affect the regulation of bone mass.

## Discussion

Regulation of bone mass via estrogen signaling has been thoroughly investigated in the past decades, with an increasing focus on the characterization of tissue-specific estrogen signaling to identify therapeutic targets for osteoporosis with minimal side effects.^14,29–31^ Previous work by us and others has discovered a tissue-specific role of membrane-initiated estrogen receptor alpha (mERα) signaling in mouse models, and shown that mERα signaling is highly involved in the regulation of bone mass.^25–28,32^ In this study, we further investigated the role of mERα signaling in specific cell types for the regulation of bone mass. The results demonstrated that mERα signaling in osteoblast lineage cells plays a crucial role in regulating female cortical bone, while mERα signaling in hematopoietic cells of adult female mice is dispensable for bone regulation.

To enable characterization of mERα signaling in specific cell types, we generated a novel conditional mERα knock-out model (C451A^f/f^). To investigate the role of mERα in osteoblast lineage cells, we used the *Runx2*-Cre mouse model, which has been reported to target osteoblast lineage cells including osteocytes, and chondrocytes, but not osteoclast lineage cells.^33^ The mutated C451A-*Esr1* gene was expressed restrictively in bone tissue of *Runx2*-C451A^f/f^ mice, showing the specificity of the model. Furthermore, the mRNA expression of total *Esr1*, including both the WT and the mutated allele, showed no difference between *Runx2*-C451A^f/f^ and control mice in cortical bone, while the expression of only the WT *Esr1* was diminished by 71% in the cortical bone tissue of *Runx2*-C451A^f/f^ mice. These data demonstrate that the overall expression of ERα is not affected by the mutation. No significant differences were found in body weight, weights of sex steroid-dependent organs or serum sex steroid concentrations between *Runx2*-C451A^f/f^ and control mice. Collectively, these data show the suitability of using *Runx2*-C451A^f/f^ mice to investigate the importance of mERα signaling in osteoblast lineage cells.

It has previously been demonstrated that loss of ERα signaling in *Runx2*-expressing cells leads to significant bone loss in spine and tibia of female mice,^34^ demonstrating that ERα signaling in osteoblasts is of importance for the regulation of bone mass. Our study shows that loss of signaling via membrane-bound ERα signaling in *Runx2*-expressing cells significantly reduces cortical bone mass and impairs mechanical strength in female mice, demonstrating that mERα signaling in *Runx2*-expressing osteoblast lineage cells is involved in the regulation of the female skeleton. Thus, the cortical bone loss previously seen after deletion of ERα in *Runx2*-expressing osteoblast lineage cells is, at least in part, caused by the loss of mERα signaling. However, nuclear estrogen receptor effects are also known to affect cortical bone mass,^14^ and the interaction between membrane-initiated and nuclear ERα actions needs further investigation.

In this study we demonstrate that mERα signaling in osteoblast lineage cells exerts a more pronounced regulatory effect on cortical bone compared to trabecular bone in long bones and vertebrae. These results are in line with data reported in a previous study by Almeida et al., using *Prx1*-Cre mice to knock out ERα signaling in osteoblast progenitors,^18^ showing decreased cortical bone thickness in female mice lacking ERα in osteoblast progenitors, while the trabecular bone mass in femur was unchanged.^18^ The results of our study demonstrated increased endosteal circumference but unchanged periosteal circumference in both femur and tibia of *Runx2*-C451A^f/f^ mice, suggesting a different regulatory mechanism from that of *Prx1*-ERαKO mice, in which both endosteal and periosteal circumference were increased.^18^ Trabecular bone mass (BV/TV) was unaffected in both femur and vertebra, while a decrease was detected in tibia. This difference implies a potential presence of distinct bone characteristics and regulatory mechanisms between the different skeletal compartments as reported previously.^35–37^

Gene expression analyses of cultured osteoblasts lacking mERα signaling demonstrated lower expression of several genes related to osteoblast differentiation (osterix, alkaline phosphatase, integrin-binding bone sialoprotein) compared to WT osteoblasts. Thus, mERα signaling in osteoblast lineage cells affects their development and maturation. Bone formation and mineral apposition rates in mice lacking mERα signaling in osteoblast lineage cells were not changed compared to controls, and these data were in line with unchanged levels of the bone formation marker P1NP in serum. The analyses of bone formation were performed in adult (10 and 14 weeks old) mice, and a difference in bone formation rate might have been detected if the analyses were performed at an earlier age. Since the inactivation of mERα signaling in osteoblasts in this model occurs early in life, when the *Runx2* promoter is activated, the exact time-point for the manifestation of the cortical bone effects is difficult to determine. The inability to establish the time when the cortical bone effects occur is a limitation of this study that warrants further investigation.

Previous studies have shown that ERα signaling in osteoclasts is important to maintain trabecular bone mass in intact mice,^16,17^ and to provide a normal response to estrogen treatment after castration.^38^ In this study, bone marrow cells from global C451A mice were transplanted into lethally irradiated adult female WT recipients to deplete mERα signaling in hematopoietic cells including osteoclasts. There were no significant differences in the evaluated bone parameters between mice receiving bone marrow from WT mice and mice receiving bone marrow from C451A mice. Thus, mERα signaling in hematopoietic cells is dispensable for the regulation of bone mass under basal conditions in adult female mice. Moreover, a previous study by Vinel et al. demonstrated that the response to estradiol treatment is similar regardless of absence of mERα signaling in hematopoietic cells in female mice,^39^ which supports our results. In order to determine the role of mERα signaling inactivation in hematopoietic cells from early life, or in specific hematopoietic cell types, experiments using Cre models such as LysM-Cre are needed. The lack of such experiments is a limitation to the current study.

In conclusion, this study shows that mERα signaling in osteoblast lineage cells is crucial for the normal regulation of cortical bone mass and mechanical strength in female mice, and that mERα signaling in hematopoietic cells of adult female mice is dispensable for normal bone regulation.

## Materials and methods

### Animals

#### Generation of a conditional C451A mERα knockout mouse model

To generate a transgenic mouse model facilitating a conditional amino acid shift (cysteine > alanine) at site 451 (C451A) in *Esr1* by introducing the mutation TGC > GCA, a targeting vector was constructed as follows (Fig. S2a): The 5’ end of the vector contained a short homology arm (SHA) targeting the intronic region before exon 7, while the 3’ end of the vector contained a long homology arm (LHA) targeting the intronic region after exon 7, leading to excision of the original exon 7 upon homologous recombination. Between the SHA and LHA, the vector contained a floxed region (FR) flanked by two loxP sites, followed downstream by exon 7 containing the C451A amino acid substitution, and the negative selection marker thymidine kinase (TK). The FR contained the following components: two positive selection markers, Neomycin resistance (NeoR) and Puromycin resistance (PuroR), flanked by the Flp recombination sites FRT and F3, respectively; the WT exons 7–9, fused without introns; and a polyadenylation signal (hGHpA cassette) to prevent downstream transcription. After Flp recombination, the floxed allele results in transcription of the fused WT exons 7–9, leading to translation of a WT ERα protein. When a Cre recombinase is introduced, the floxed region is excised and the exon 7 containing the mutation is instead transcribed, leading to translation of the mutated ERα C451A protein. The targeting vector was generated using BAC clones from the C57BL/6J RPCI-23 BAC library and was transfected into the Taconic Biosciences C57BL/6N TacES cell line. Homologous recombinant clones were isolated using double positive (NeoR and PuroR) and negative (TK) selections. The generation of this transgenic mouse model (C57BL/6NTac-Esr1^tm6116(C451A)Tac^) was conducted at Taconic (Borup, Denmark), and is denoted as C451A^f/f^ mice.

To verify that the introduction of the floxed region does not affect the phenotype, homozygous C451A^f/f^ female mice were compared to WT littermates at 16 weeks of age. The C451A^f/f^ female mice manifested the same phenotype as WT female littermates regarding organ weights, serum steroid concentrations, and bone parameters (Table S2).

#### Generation of a global C451A mERα knockout mouse model

To generate mice with a global ERα C451A mutation (C451A mice), that lack mERα signaling in all cells, *Pgk1*-Cre mice were used, targeting both germ cells and somatic cells.^40^ C451A^f/f^ mice were bred with *Pgk1*-Cre mice, and insertion of the mutation was confirmed by DNA sequencing of ear clips from the C451A mice (*n* = 3) and WT littermates (*n* = 3) (Eurofins Genomics, Ebersberg, Germany), validating the C451A^f/f^ model (Fig. S2b). The C451A mice, generated using *Pgk1*-Cre mice, exhibited disturbed serum steroid levels and unaffected skeletal bone mass compared to WT controls (Table S3). These results are in line with the phenotypes shown in C451A-ERα mice generated by knock-in mutation.^22,28,39^ C451A female mice and their WT female littermates were used as donors for the bone marrow transplant (BMT) experiment and in the in vitro experiments (see below).

#### Generation of an osteoblast lineage-specific C451A mERα knockout mouse model

To generate mice with specific inactivation of mERα signaling in osteoblast lineage cells, C451A^f/f^ mice were bred with *Runx2*-Cre mice, generating Runx2-C451A^f/f^ mice. It has been shown previously that *Runx2*-Cre mice manifest the same skeletal phenotype as WT littermates.^41^ *Runx2*-C451A^f/f^ female mice were used to study the effects of lacking mERα signaling in osteoblast lineage cells, and homozygous C451A^f/f^ female littermates were used as controls. Two independent studies were conducted, one terminated at 10 weeks, and one terminated at 14 weeks of age (*n* = 4–7 per group).

All animals were kept in a standard animal facility with regulated temperature (22 °C) and a 12-hour light:12-h darkness cycle. Mice were provided with a phytoestrogen-free pellet diet (Teklad diet 2016, Envigo, Indianapolis, Indiana, United States) and tap water *ad libitum*. Animal experiments were approved by the Ethical Committee for Animal Research in Gothenburg (Göteborgs djurförsöksetiska nämnd) and reported according to ARRIVE guidelines. Primers used for genotyping the mice are listed in Table S4.

At termination, mice were anesthetized with Ketador/Dexdomitor (Richter Pharma, Wels, Austria/Orion Pharma, Espoo, Finland). Blood samples were obtained from the axillary artery, followed by euthanasia through cervical dislocation. Soft tissues were dissected, weighed, snap-frozen in liquid nitrogen, and stored at −80 °C. Bone marrow was collected from tibia and femur by cutting the ends followed by centrifugation, snap-frozen in liquid nitrogen, and stored at −80 °C. The remaining cortical bone was collected from tibia and femur and stored at −80 °C for DNA isolation or RNA preparation (stored in RNAprotect Tissue Reagent, Qiagen, Hilden, Germany). Tibia, femur, and vertebra L5 were dissected, fixed in 4% paraformaldehyde for 2 days and then stored in 70% ethanol for further analyses. One vertebra L5 in the 10-week-old *Runx2*-C451A^f/f^ group was excluded because of dissection damage. Humerus was dissected and stored at −20 °C for three-point bending analysis.

### Bone marrow transplantation

Wildtype C57BL/6NTac female mice were lethally irradiated with a RS 2000 X-ray irradiator (Rad Source Technologies, Georgia, USA). The radiation dosage administrated was 4.5 Gy over a duration of 170 s per session, repeated twice with a four-hour interval of rest, reaching a total dosage of 9 Gy. After the second irradiation, BMT was performed by intravenous injection of 350 000 donor bone marrow cells obtained from either C451A female mice, which lack mERα signaling in all cells, or WT female littermates. The donor cells were purified before transplantation using EasyStep^TM^ Mouse Hematopoietic Progenitor Cell Isolation Kit (STEMCELL Technologies, Vancouver, Canada). WT recipient mice receiving WT bone marrow (WT/WT) were used as controls for WT recipient mice receiving C451A bone marrow (WT/C451A). Antibiotic treatment with 0.6 mg/mL Baytril (Bayer, Leverkusen, Germany) in the drinking water was started one week before BMT and discontinued two weeks after BMT. All mice received radiation and BMT at 11 weeks of age, and they were sacrificed at 16 weeks of age. Since the mice were part of another experiment (unpublished data), they were sham operated at 10 weeks of age and received subcutaneous injections of vehicle treatment (Miglyol 812, OmyaPeralta GmbH, Hamburg, Germany) once every second day for three weeks before termination. The efficiency of cell replacement after irradiation and BMT was determined in a separate experiment, with flow cytometer analysis, showing that 79.5% ± 10.8% (SD) of the bone marrow cells were of donor origin 12 weeks post-BMT (for detailed method description see Fig. S3).

### Serum analyses

Circulating concentrations of serum E2 and T were quantified using high-sensitivity liquid chromatography-tandem mass spectrometry (LC–MS/MS) as described previously.^42^ The lower limit of quantification (LLOQ) of LC-MS/MS is 0.5 pg/mL for E2 and 5 pg/mL for T. Sex steroid concentrations below LLOQ were used as half of LLOQ (0.25 pg/mL for E2 and 2.5 pg/mL for T) in the statistical analyses. As a marker of bone formation, serum levels of procollagen type I N propeptide (P1NP, Immunodiagnostic Systems, Copenhagen, Denmark) were analyzed by ELISA and as a marker for bone resorption, terminal type I collagen fragments were measured in serum using an ELISA RatLaps kit (CTX-I, Immunodiagostic Systems).

### DNA and RNA isolation, and real-time PCR

DNA was isolated from tissues using DNeasy Blood & Tissue Kit (Qiagen). Total mRNA from cortical bone was isolated using TriZol (Life Technologies, Thermo Fisher Scientific, Waltham, MA) and RNeasy Mini Kit (Qiagen). Total mRNA from soft tissues was isolated using RNeasy Mini Kit. The isolated mRNA was reversely transcribed into complementary DNA (cDNA) using the High-Capacity cDNA Reverse Transcription kit (Applied Biosystems, Thermo Fisher Scientific). Real-time PCR amplification was performed by Applied Biosystem StepOnePlus Real-Time PCR System (Thermo Fisher Scientific). The Assay-on-Demand primer and probe sets (Thermo Fisher Scientific) used in this study included Estrogen receptor alpha (*Esr1*: Mm00433147_m, targeting a cDNA region upstream of exon 6, thereby detecting both the WT and the mutated *Esr1* expression) and 18S (4310893E), which was used for normalization. A customized assay (Thermo Fisher Scientific) was used to quantify the relative DNA abundance of the C451A-Esr1 gene: C451A F: GCCCAACCACACAGTCCATA, C451A R: TCTCCTCCTGATGTGTCTTGA, with FAM-MGB fluorescent dye to detect the C451A mutated DNA allele, and for normalization, an intronic region (Ctrl) upstream of the LoxP sites was quantified using the following primers with VIC-MGB fluorescent dye: Crtl F: CACATGACTGCTGGGCATTT, Ctrl R: AATGCACGTATGAGCACTGG. The relative RNA expression of WT *Esr1* was measured by using PowerUp^TM^ SYBR^TM^ Green Master Mix (Applied Biosystems) and customized primers targeting the WT codon bases TGC at site 451 of *Esr1* (Applied Biosystems, F: GGAAGCTCCTGTTTGCTCCT, R: GCAAAATGATGGATTTGAGGCA) normalized to the expression of *Ppia3* (PrimerBank-MGH-PGA ID: 6679439a1, Applied Biosystems). The relative abundance of target sequences was normalized to either 18S, *Ppia3* or Ctrl, and calculated using the ΔΔCt method.

### Peripheral quantitative computed tomography

Peripheral quantitative computed tomography (pQCT) was performed with the pQCT XCT RESEARCH M (version 4.5B, Norland, UK) operating at a resolution of 70 μm as described before.^43^

#### Femur

Cortical bone parameters were analyzed in the mid-diaphyseal region at a distance proximal from the distal growth plate corresponding to 36% of the total length of the femur. The scan for trabecular bone parameters was positioned in the metaphysis of the femur at a distance proximal from the distal growth plate corresponding to 3% of the total length of the femur. The trabecular bone region was defined as the inner 45% of the total cross-sectional area.

#### Tibia

Cortical bone parameters were analyzed in the mid-diaphyseal region at a distance distal from the proximal growth plate corresponding to 30% of the total length of the tibia. The scan for trabecular bone parameters was positioned in the metaphysis of the tibia at a distance distal from the proximal growth plate corresponding to 2.6% of the total length of the tibia. The trabecular bone region was defined as the inner 45% of the total cross-sectional area.

### High‐resolution microcomputed tomography

High-resolution microcomputed tomography (μCT) analysis was performed on the femur, tibia, and vertebra L5 using the Skyscan 1275 model (Bruker MicroCT, Billerica, Massachusetts, United States), with an X-ray tube voltage of 40 kV and a current of 200 μA. The angular rotation was set at 180°, with an angular increment of 0.40°. Voxel size was isotropically maintained at 7 μm.

#### Femur

Cortical parameters of femur were assessed in the diaphyseal region, initiating 5.2 mm from the distal growth plate, and extending longitudinally for 210 μm in the proximal direction. The trabecular bone analysis focused on the region proximal to the distal growth plate, within a defined volume of interest excluding cortical bone. The analysis of trabecular bone started 504 μm from the growth plate and extended a further longitudinal distance of 210 μm in the proximal direction.

#### Tibia

Cortical parameters of tibia were assessed in the diaphyseal region, initiating 5.2 mm from the proximal growth plate, and extending longitudinally for 210 μm in the distal direction. The trabecular bone analysis focused on the region distal to the proximal growth plate, within a defined volume of interest excluding cortical bone. The analysis of trabecular bone started 504 μm from the growth plate and extended a further longitudinal distance of 210 μm in the distal direction.

#### Vertebra

Analysis of cortical and trabecular parameters of L5 were initiated 7 µm caudal of the lower end of the pedicles, extending a further longitudinal distance of approximately 245 µm in the caudal direction. The region of interest for cortical and trabecular bone were manually drawn and analyzed using CTAn software.

### Three-point bending

The three-point bending was performed with a span length of 5.5 mm and a loading speed of 0.155 mm/s was applied to the humerus using an Instron 3366 (Instron, Norwood, Massachusetts, United States). Biomechanical parameters were derived from the load deformation curves using Bluehill Universal software version 4.25 (Instron), and calculations were performed using Excel (Microsoft).

### Dynamic histomorphometry

For dynamic histomorphometry, mice were administered intraperitoneal injections of the fluorochromes calcein and alizarin (Merck GmbH, Darmstadt, Germany) 9 days and 2 days prior to sacrifice, respectively. Following dissection, the femurs were fixed in 4% formaldehyde for 48 h, dehydrated using a graded ethanol series, and embedded in LR White Resin (London Resin Co, Ltd, UK). The femurs were sectioned transversely at the mid-diaphysis into 200-µm-thick slices. The slices were ground down to a single-cell layer of approximately 7 µm thickness using 800, 1 200, and 4 000 grit silicon carbide paper (Struers A/S, Rødovre, Denmark) with the aid of an automatic grinder (EXAKT® cutting and grinding equipment, EXAKT® Apparatebau GmbH & Co, Norderstedt, Germany). The dynamic cortical bone parameters were evaluated without any additional staining by using the Bioquant OSTEO software version 2023.v23.5.60 (Bioquant Image Analysis Corporation, Heidelberg, Germany) according to the American Society for Bone and Mineral Research guidelines.^44^

### Primary osteoblast culture

Primary periosteal bone cells were isolated from calvariae of 3–5 days old global C451A mice and WT littermate controls by sequential enzymatic digestion.^45^ 5–7 dissected calvariae per genotype were used in each experiment. The calvariae were washed in PBS after dissection and incubated in 5 mL 4 mmol/L EDTA in PBS at 37 °C, 600 r/min rotation table for 2 sequential 10-min digestions, followed by 7 sequential 10-min digestions of 5 mL 180 U/mL Collagenase type II (BioNordika, Worthington, Ohio, United States) in PBS. The first two collagenase fractions were discarded and the last five were pooled. Thereafter, the primary calvarial periosteal bone cells were cultured in complete α-MEM medium (Gibco, Thermo Fisher Scientific) supplemented with 10% heat-inactivated fetal bovine serum (Sigma), 2 mmol/L GlutaMAX (Gibco), 50 µg/mL gentamicin (Gibco), 100 U/mL penicillin and 100 µg/mL streptomycin (Gibco) for 3–5 days prior to the experiments. At the start of experiments, cells were detached with trypsin and re-seeded at approximately 18 000 cells/cm^2^ in osteogenic media (complete α-MEM medium supplemented with 4 mmol/L β-glycerophosphate, Sigma, and 0.28 mmol/L L-Ascorbic acid 2-phosphate sesquimagnesium salt hydrate, Sigma).

#### RNA isolation and real-time PCR

At the point of harvesting RNA (after 1, 4, and 7 days), cultured cells were lysed in RNeasy lysis (RLT) buffer with beta-mercaptoethanol (Qiagen). Total mRNA was isolated using RNeasy Micro Kit (Qiagen). cDNA reverse transcription and real-time PCR were performed and analyzed as described above. The Assay-on-Demand primer and probe sets (Thermo Fisher Scientific) used included alkaline phosphatase (*Alpl*: Mm00475834_m1), Sp7 transcription factor 7 (*Sp7*/Osterix/*Osx*: Mm04209856_m1), integrin binding sialoprotein (*Ibsp*: Mm00492555_m1), and 18S (4310893E), which was used for normalization.

#### Alkaline phosphatase (ALP) staining

At day 7, the cells were fixed by citrate buffered acetone and stained according to the commercial kit protocol (85L2-1KT, Sigma). In short, cells were fixed for 30 s and washed three times with distilled water (dH2O), followed by 20 min of ALP staining and three washes with dH2O. The photo for quantification was taken with ChemiDoc XRS+ (Bio-Rad, Hercules, California, United States) and analyzed by Image Lab 6.1.

All in vitro studies were repeated three times with similar results.

### Statistical analysis

Statistical analysis and figure visualization were conducted using GraphPad Prism (version 10.0.3), while table analyses were carried out using Microsoft Excel (version 16.79.2). Values in the figures are displayed as individual values with mean (horizontal line) and SEM (vertical line), while tables present values as mean ± SD. For normally distributed continuous data, differences between two independent groups were assessed using Student’s *t* test (Excel). For data without normal distribution, the non-parametric two-tailed Mann–Whitney *U* test was applied to evaluate differences between two independent groups (GraphPad Prism). Two-way ANOVA was applied to examine the influence of two independent variables (GraphPad Prism), and Šidák´s multiple comparisons test was used to compare the difference between genotypes within each time point. In all conducted analyses, a difference was considered significant when *P* < 0.05.

## Supplementary information

Yiang et al. Supplemental data

## Data Availability

The data supporting the results of this study are available on request from the corresponding author.
