# Supplementary material for: Membrane-initiated estrogen receptor-α signaling in osteoblasts is crucial for normal regulation of the cortical bone in female mice
Source: Bone Res. 2025 Jun 17;13:65. doi: 10.1038/s41413-025-00439-8 (PMC12170823; doi:10.1038/s41413-025-00439-8)
Supplement: Supplementary file 1 — Yiang et al. Supplemental data [file 41413_2025_439_MOESM1_ESM.pdf]

Supplementary material for:

Membrane-initiated Estrogen Receptor-α Signaling in Osteoblasts is Crucial for Normal Regulation of the Cortical Bone in Female Mice

Yiwen Jiang[1#], Karin Horkeby[1#], Petra Henning[1], Jianyao Wu[1], Karin H Nilsson[1], Lina Lawenius[1], Sofia Movérare-Skrtic[1], Priti Gupta[1,3], Cecilia Engdahl[1,3], Antti Koskela[4], Juha Tuukkanen[4], Lei Li[1], Claes Ohlsson[1,2], Marie K Lagerquist[1]

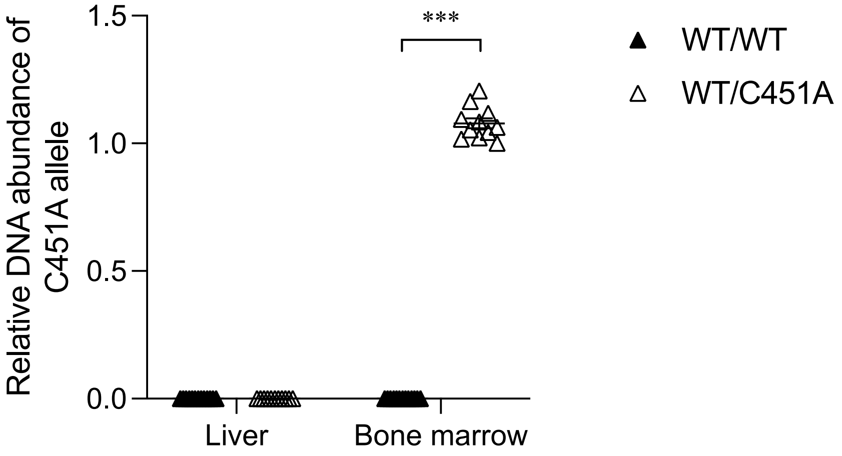

Supplementary Figure 1. DNA analysis of the relative abundance of the C451A allele in liver and bone marrow from wildtype (WT) recipient female mice receiving WT bone marrow (WT/WT) and WT recipient female mice receiving C451A bone marrow (WT/C451A) showed specific detection of the mutated C451A allele in the bone marrow of WT/C451A female mice. All individual values are presented with mean (horizontal line) and SEM (vertical lines). Mann-Whitney Test was applied. ***$p<0.001$ vs WT/WT, n=11-13.

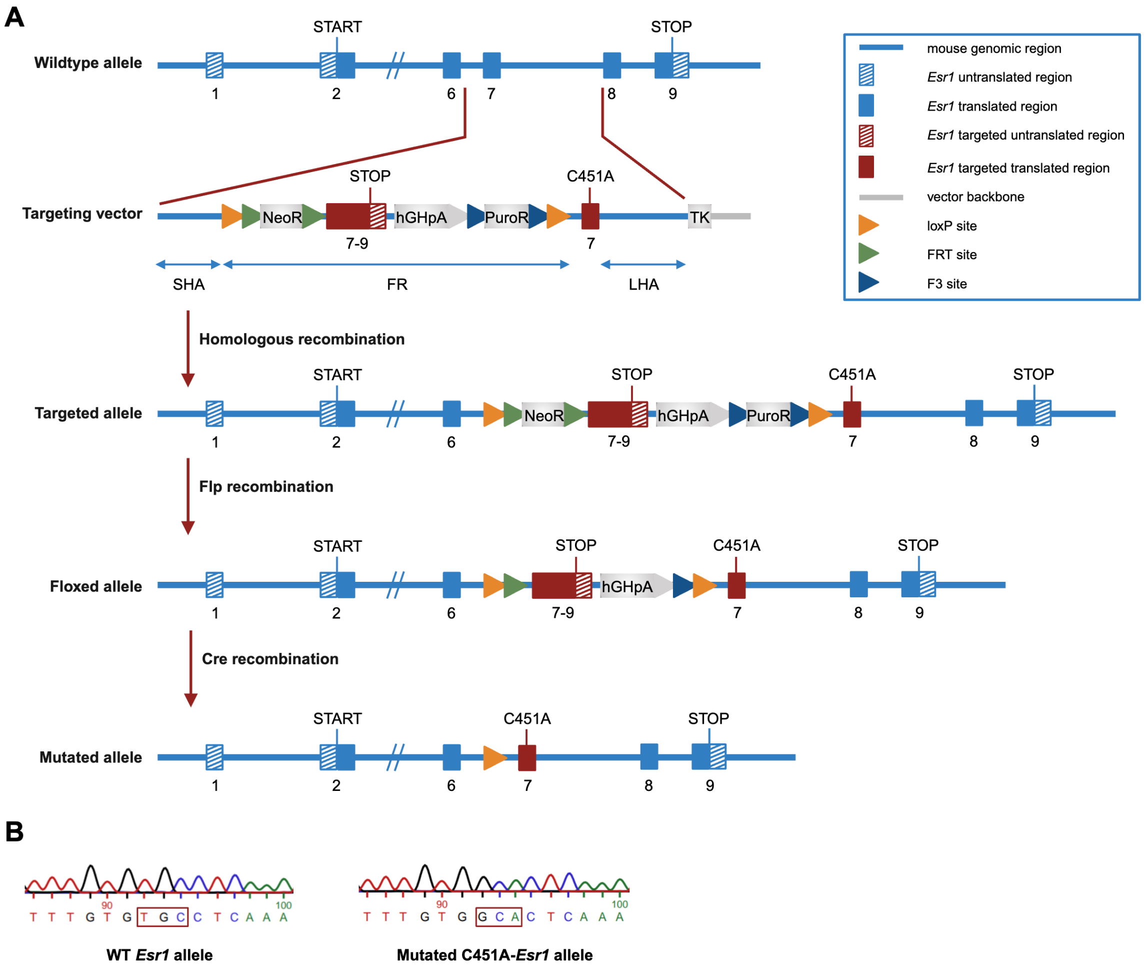

Supplementary Figure 2. Generation of a mouse model with a conditional mutation of C451A-*Esr1* based on the Cre-LoxP recombination system.

A targeting vector was constructed as shown in (A), and after homologous and Flp recombination, a floxed allele was achieved. Mice with a homologous floxed allele (C451A$^{f/f}$) express wildtype (WT) ERα protein and when a Cre is introduced, the mutated C451A ERα protein is expressed in Cre-positive cells. (B) DNA sequencing of a C451A mouse and a WT littermate control, generated by breeding C451A$^{f/f}$ with *Pgk1*-Cre, confirmed the amino acid shift of cysteine (TGC) to alanine (GCA) at site 451 in *Esr1*. SHA; short homology arm, FR; floxed region, LHA; long homology arm.

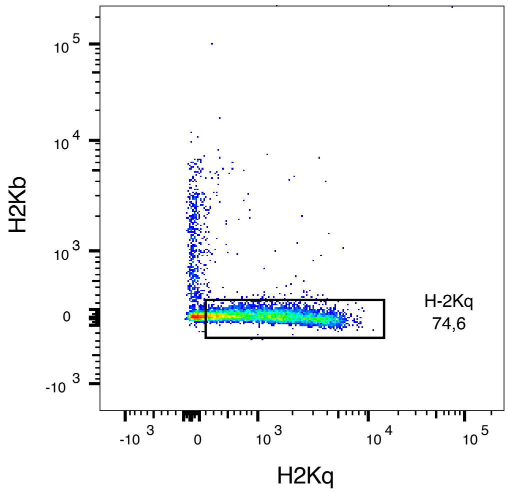

Supplementary Figure 3. Confirmation of successful cell replacement after irradiation and bone marrow transplant.

Representative figure from flow cytometry analysis. C57BL/6N female mice were irradiated (two sessions of 4.5 Gy, as described in the method section) at eight weeks of age and received bone marrow from DBA/1 mice (Janvier, France). Two weeks after bone marrow transplant (BMT), the mice were ovariectomized. Twelve weeks after BMT, flow cytometry analysis of blood cells was performed. Since the mouse strains used have different MHC-I class molecules (C57BL/6 mice express H-2Kb and DBA/1 mice express H-2Kq), the bone marrow cells from each strain can be identified using antibodies. Blood was collected, mixed with 0.5 M EDTA, washed in PBS, and centrifuged. The cell pellet was resuspended in Tris-buffered 0.83% NH_4Cl solution to lyse erythrocytes, washed twice in PBS, resuspended in FACS buffer [PBS supplemented with 10% FCS (Thermo Fisher) and 0.1% NaN3], and counted using an automated cell counter (Sysmex Europe GmbH, Norderstedt, Germany). The cells were stained with Fc-γ receptor block, PE-conjugated anti-H-2Kb (clone AF6-88.5, BioLegend, San Diego, CA, USA), and FITC-conjugated anti-H-2Kq (clone KH 114, BioLegend). Samples were run on a FACSVerse (Becton Dickinson, Franklin Lakes, USA) and FlowJo software version 10.10.0 (Tree Star, Ashland, USA) was used for data analysis. The result shows that 12 weeks after BMT 79.5% ± 10.8 SD (n=3) of the bone marrow cells were of donor origin, confirming a successful replacement of bone marrow cells after radiation and BMT.

Supplementary table 1. Measurement of bone parameters in the tibia and vertebra L5 of female mice lacking mERα signaling in hematopoietic cells.

		WT/WT	WT/C451A
Bone parameters	Tibia Tb. BMD (mg/cm^3)	223 ± 43	237 ± 37
	Tibia Ct. Th. (μm)	189 ± 12	195 ± 8
	L5 BV/TV (%)	17.5 ± 2.0	19.3 ± 2.3
	L5 Ct.Th. (μm)	67.2 ± 4.2	71.3 ± 5.3

Bone parameters of wildtype (WT) recipient mice receiving WT bone marrow (WT/WT, n=13) and WT mice receiving C451A bone marrow (WT/C451A, n=10). Tibia measured by peripheral quantitative computed tomography and vertebra L5 by high-resolution microcomputed tomography. Student's *t*-test was applied. All values are presented as mean ± SD. Tb. BMD; trabecular bone mineral density, Ct. Th.; cortical thickness, BV/TV; trabecular bone volume per tissue volume.

Supplementary Table 2. Homozygous ERα C451A$^{f/f}$ female mice manifest the same phenotypes as wildtype female littermates.

		**Wildtype**	**C451A$^{f/f}$**
Organ weights	Body weight (g)	20.8 ± 0.5	20.8 ± 1.4
	Uterus weight/BW (mg/g)	4.5 ± 1.8	3.1 ± 0.7
	Liver weight/BW (mg/g)	41.9 ± 4.5	42.7 ± 4.5
	Thymus weight/BW (mg/g)	3.2 ± 0.5	3.0 ± 0.5
	Gonadal fat weight/BW (mg/g)	11.0 ± 3.0	14.5 ± 6.1
Bone parameters	Tibia Tb. BMD (mg/cm^3)	270 ± 32	276 ± 51
	Tibia Ct. Th. (μm)	190 ± 11	186 ± 11
	Femur Tb. BMD (mg/cm^3)	374 ± 31	364 ± 61
	Femur Ct. Th. (μm)	177 ± 8	183 ± 10
	Vertebra L5 BV/TV (%)	19.0 ± 2.4	20.1 ± 2.5
Sex steroids	Estradiol (pg/mL)	3.5 ± 4.3	2.3 ± 1.2
	Testosterone (pg/mL)	15.3 ± 13.4	20.2 ± 4.5

Homozygous ERα C451A$^{f/f}$ female mice and wildtype (WT) female littermate controls were examined at 16 weeks of age. Tibia and femur bone mass was measured by peripheral quantitative computed tomography and vertebra L5 by high-resolution microcomputed tomography. WT (n = 6) and C451A$^{f/f}$ (n = 9). Serum concentrations of estradiol and testosterone were measured by high-sensitivity liquid chromatography-tandem mass spectrometry in WT (n = 4) and C451A$^{f/f}$ (n = 5). Student's *t*-test was applied to analyze the differences for organ weights and bone parameters. The Mann-Whitney U test was applied to analyze the differences for serum sex steroids. All values are presented as mean ± SD. BW; body weight, Tb. BMD; trabecular bone mineral density, Ct. Th.; cortical thickness, BV/TV; trabecular bone volume per tissue volume.

Supplementary Table 3. Global ERα C451A female mice show disturbed serum steroid levels.

		Wildtype	C451A
Organ weights	Body weight (g)	21.8 ± 0.8	21.8 ± 1.1
	Uterus weight/BW (mg/g)	3.0 ± 0.9	3.8 ± 1.0
	Liver weight/BW (mg/g)	44.8 ± 5.7	43.2 ± 3.6
	Thymus weight/BW (mg/g)	2.6 ± 1.1	2.1 ± 0.7
	Gonadal fat weight/BW (mg/g)	15.0 ± 4.1	18.7 ± 4.0
Bone parameters	Tibia Tb. BMD (mg/cm^3)	285 ± 25	272 ± 45
	Tibia Ct. Th. (μm)	183 ± 5	193 ± 11
	Femur Tb. BMD (mg/cm^3)	382 ± 77	338 ± 57
	Femur Ct. Th. (μm)	182 ± 9	182 ± 7
	Vertebra L5 BV/TV (%)	18.1 ± 1.4	16.1 ± 2.0
Sex steroids	Estradiol (pg/mL)	0.5 ± 0.5	15.2 ± 14.1**
	Testosterone (pg/mL)	13.9 ± 7.8	84.1 ± 81.2**

Global ERα C451A female mice (n=6) and wildtype (WT) littermate controls (n=7) were examined at 13 weeks of age. Tibia and femur bone mass was measured by peripheral quantitative computed tomography and vertebra L5 by high-resolution microcomputed tomography. Serum concentrations of estradiol and testosterone were measured by high-sensitivity liquid chromatography-tandem mass spectrometry. Student's *t*-test was applied to analyze the differences for organ weights and bone parameters. The Mann-Whitney U test was applied to analyze the differences for serum sex steroids. All values are presented as mean ± SD. ***P* < 0.01, BW; body weight, Tb. BMD; trabecular bone mineral density, Ct. Th.; cortical thickness, BV/TV; trabecular bone volume per tissue volume.

Supplementary Table 4. Primers used for genotyping.

Breeding	Function	Direction	Sequence (5′-3’)
Pgk1-Cre X C451A$^{f/f}$	To detect *Pgk1*-Cre allele	Forward	GTTCGCAAGAACCTGATGGACA
		Reverse	CTAGAGCCTGTTTTGCACGTTC
	To distinguish between mutated C451A-*Esr1* allele and wildtype *Esr1* allele	Forward	TTGAACCCTGACTTTCTCGG
		Reverse	ACCCGACATTAAATTCCCC
Runx2-Cre X C451A$^{f/f}$	To distinguish between *Runx2*-Cre allele and wildtype *Runx2* allele	Forward	CCAGGAAGACTGCAAGAAGG
		Reverse	TGGCTTGCAGGTACAGGAG
		Reverse	GGAGCTGCCGAGTCAATAAC
	To detect the floxed allele	Forward	TTGAACCCTGACTTTCTCGG
		Reverse	TTCAAGGTGCTGGACAGAAAC
	To detect wildtype *Esr1* allele	Forward	CCACACAGTCCATATCTGCTAGA
		Reverse	TGTTGAATGTGGAGATCTGTGG
